# Health Risk Assessment of Informal Food Vendors: A Comparative Study in Johannesburg, South Africa

**DOI:** 10.3390/ijerph20032736

**Published:** 2023-02-03

**Authors:** Maasago Mercy Sepadi, Vusumuzi Nkosi

**Affiliations:** 1 Department of Environmental Health, Faculty of Health Sciences, Doornfontein Campus, University of Johannesburg, Johannesburg 2094, South Africa; 2 Environment and Health Research Unit, South African Medical Research Council, Johannesburg 2094, South Africa; 3 Faculty of Health Sciences, School of Health Systems and Public Health, University of Pretoria, Pretoria 0001, South Africa

**Keywords:** street vendors, air pollution, respiratory, prevalence, risk assessment, occupational health, environmental health

## Abstract

According to the best of our knowledge, there are no critical studies to date about associations between the work environment and prevalence of respiratory diseases and their symptoms among urban informal vendors in South Africa. This study followed a risk assessment methodology to assess the risks associated with the occurrence of respiratory health problems among 617 indoor and outdoor market vendors in the inner city of Johannesburg, South Africa. A walkthrough survey using a checklist was conducted in 16 Markets for identification of respiratory risk factors and description of sanitary conditions. Face-to-face interviews were conducted amongst informal food vendors using a previously validated questionnaire to collect information on demographics and occupational and respiratory health. In addition, a single day area air pollution monitoring of PM_2.5_, SO_2_, NO_2_, CO, and CO_2_ was conducted in two stalls (indoor and outdoor). The Chi-squared test of association and frequency distribution were used to analyse data. Female vendors aged between 30 to 39 years dominated the trade. The results revealed that a majority of the vendors’ work shifts are longer than the recommended eight hours per day (73%), six to seven days per week (90%), and most of them have been working for six to ten years (41%). Poor sanitary conditions were observed in 75% of the markets. The concentrations of air pollutants at the outdoor markets were much greater than those in the indoor markets. All air pollution concentrations were below the recommended national and international standards. The risk of outdoor vendors developing any symptoms were extremely greater than those of indoor vendors, especially cooking vendors. Upper respiratory symptoms were the highest amongst the vendors. The results demonstrated a significant association between work-related risk factors, such as type of work location, duration, type of cooking fuel or heat, vendor training, frequency of hand hygiene practice, and using of a mask, and the upper respiratory symptoms. Based on the findings, there is a need for formalization of the trade, improvement in infrastructure, respiratory health care assessments, and sustainable educational programs.

## 1. Introduction

The work environment contributes to the health of workers in general. An exposure evaluation of any workplace must take into account surrounding risk factors, sources and emissions variables, pathways, and routes of intake or uptake. Working conditions for informal vendors are characterized by low wages, the absence of social security or state benefits, long working hours, and hazardous workplace environments [1]. According to research conducted in South Africa, the workstations of informal vendors are characterized by a lack of infrastructure, poor general cleanliness, a lack of compliance with personal protective equipment (PPE) [2,3,4,5], and exposure to ambient air pollution [6].

Air pollution is the most relevant environmental cause of disease and premature death in the world today. With various emissions coming from work activities and the surrounding sources; inhalation was reported by research as the fastest way to absorb substances, followed by skin contact and ingestion [6]. Moreover, the way that a substance is absorbed can have a big impact on how it affects your health [7,8,9,10].

All major organs may be impacted by ambient air pollution. Fine particulate matter (PM_2.5_) causes conditions that affect the cardiovascular and respiratory systems, including lung cancer, stroke, and chronic obstructive pulmonary disease (COPD) [7,8]. Sulphur dioxide (SO_2_) and nitrogen dioxide (NO_2_) exposures was associated with increase in respiratory infections such as breathing difficulties, particularly for people with asthma, heart disease, and airway inflammation [7,8]. The exacerbation symptoms of heart disease, vision problems, and the reduction in physical and mental capabilities in healthy people was associated with exposure to carbon monoxide (CO), and carbon dioxide (CO_2_) poisoning may result in death [7,8].

Environmental and occupational risk factors related to ambient air pollution have been reported to cause acute and chronic respiratory illnesses, such as increased rates of acute upper respiratory tract infections (eye, nose, and throat irritation), which may interfere with the work operations [7,11,12,13,14,15] and reproductive health impact of female vendors [10,11]. Studies conducted in Ghana, Bangkok, Malaysia, and Hong Kong reported that the outdoor vendors are a high-risk group for developing respiratory symptoms due to continuous exposure to the traffic pollutants, as well as the particulate matter arising from the use of fossil fuels during cooking [12,13,14,15]. The risk of respiratory infections including the flu, colds, and COVID-19 amongst informal vendors may also be increased by contaminated hands during working hours [16] and not wearing PPE [17]. Furthermore, these exposures and health problems may be impacted by longer working hours. Standard or normal working hours should not exceed forty-eight hours per week and eight hours per day, according to ILO Conventions 1 and 30 [18]. However, vendors have been known to work more than 8 h a day. The health system is currently under threat due to the impact of chronic illnesses; it has been reported that chronic respiratory diseases are an economically relevant public health problem in all countries [19]. Furthermore, it has been acknowledged that air pollution and its contribution to ill-health is a serious sustainability issue and that it has an impact on the Sustainable Development Goals [20].

The study’s target population is one of the understudied subfields of environmental and occupational health assessment and intervention implementation in South Africa. In the recent study, we compared the results of the population of indoor and outdoor food vendors in the inner city of Johannesburg in order to evaluate the effects of air pollution and related risk factors on the respiratory health of informal food vendors [9].

## 2. Materials and Methods

This cross-sectional analytical study was conducted in the inner city of Johannesburg, South Africa [9]. A sample size of 617 informal vendors from 16 markets participated in the study. The study methodology was designed according to the steps of the human health risk assessment.

### 2.1. Hazard Assessment

Through the database of the inner city of Johannesburg, 16 markets or demarcated sites participated in the study [9]. A preliminary walkthrough survey or observations of the vendors’ markets was conducted using a checklist [9] for identification of respiratory risk factors and description of sanitary conditions of the 16 vendor markets. 

### 2.2. Exposure Assessment

PM_2.5_, CO, CO_2_, NO_2_, and SO_2_ area or static monitoring was conducted. The air sampling was conducted in winter season on the 30 June 2022 between 8 am and 3 h 30 pm. For ambient concentrations of PM_2.5_, the GilAir pump was used to collect samples, an EXTECH air quality monitor for CO and CO_2_, and the Radiello passive sampler was used to monitor the NO_2_ and SO_2_. The static air samplers were placed in one stall in each market (outdoor and indoor stall) at 1.5 m height [9]. The samples were analyzed at a SANAS 17025 accredited laboratory.

Face-to-face interviews of 617 vendors were conducted using a previously validated structured researcher-administered questionnaire, collecting demographic and operational information and self-reported respiratory symptoms and diseases in the last 12 months [9]. The questionnaire was administered between May and August 2022, and interviews were conducted at each vendor’s stall during operating hours [9].

### 2.3. Risk Characterization and Consequences Assessment

The measured levels of air pollutants were compared to the South African and international standards which are used as evidence based recommended limits. The South African Regulations for hazardous chemical agents, 2021 (Act No. 280 of 2021), was used as guidance for PM_2.5_, CO, and CO2 [21]. The identified occupational exposure limits were 5 mg/m^3^ for PM_2.5_, 4000 ppm for CO, and 5000 ppm for CO_2_, averaged over an 8 h workday. The South African National Environmental Management: Air Quality Act, 2004 (Act No. 39 of 2004) was used as guidance for NO_2_ (200 μg/m^3^ for an hour) and SO_2_ (125 μg/m^3^ for 24 h) standards [22]. The following values and interim targets are recommended by the WHO (2021) Air quality guidelines on 24 h average time; 15 μg/m^3^ for PM_2.5_, 40 μg/m^3^ for SO_2_, 4 μg/m^3^ for CO [23], and a short average exposure of an hour of 200 μg/m^3^ for NO_2_ [24]. The National Institute for Occupational Safety and Health (NIOSH) recommended the exposure limit for CO_2_ is 5000 ppm for an 8 h workday [25]. The SPSS tool was used for analysis. Estimation of the prevalence of respiratory symptoms and diseases in relation to the numbers of the population were reported in frequencies. A chi-squared test was conducted between the respiratory symptoms and demographic and operational characteristics such as work location (indoor and outdoor) and type of vendor (cooking and non-cooking); and *p*-values were considered significant at *p* < 0.05.

## 3. Results

### 3.1. Hazard Assessment

#### 3.1.1. Vendor Market Description

This study included a total of 16 markets or vending locations. Markets in discrete locations (inside buildings/in enclosed permanent brick structure) accounted for (n = 7; 44%). While outdoor markets accounted for 56% (n = 9). Approximately four (25%) of the markets were at a transportation hub stop, e.g., taxi rank, railway, and bus station, while the remaining were in designated market premises with no other activities taking place. 

Since the study was conducted in Johannesburg’s inner city, the vendor stalls surrounding visuals included residential flats, office buildings, shops, industrial premises, and offensive trades. Furthermore, the markets were near high traffic density due to transportation hubs within the city; the internal inner city intensive automotive movement was occurring throughout the day, especially in the mornings and afternoon. Moreover, the city is also close to M1 south and north highways, which normally have heavy traffic congestion.

#### 3.1.2. Hazard Identification

The five investigated ambient air pollutants were PM_2.5_, which is in the nature of solid particles, SO_2_, NO_2_, CO, and CO_2_, which are gases. For this study, inhalation was the focus in order to identify the respirable health impact amongst informal food vendors. Traffic or automotive related air pollutants are a main concern of working along the road, which was also found to be affecting vendors [9,10]. The other identified sources of exposure included road dust, construction sites, industrial processes, poorly ventilated small stall spaces, dusty vendor working areas, use of cooking fuels such as gas, and open fires.

In terms of sanitary conditions in vendor markets, lack of infrastructure was noted during the walkthrough survey. Shelter or building protection against environmental nuisances was provided only in seven markets, all of which were indoors, and one of which was outdoors. There were poor sanitary conditions observed in 12 of the markets (75%) and only 4 (25%) of the markets were in good sanitary conditions. In and around the 16 respective markets or vending sites, the following sanitary facilities were observed: central waste storage area or dumpsite (n = 9; 56%); on-site water points or facilities (n = 10; 63%); wash hand basins (n = 9; 56%), hand drying facility (n = 3; 19%), and hand wash-soaps (n = 3; 19%). Flies (n = 11; 69%) were also present, as were rats or cockroaches (n = 12; 75%) and visible dust (n = 13; 81%).

### 3.2. Informal Food Vendor’s Demographic Information

According to this study, there is a slight difference in nationality among vendors, with South Africans accounting for 54%, compared to 46% of non-South Africans (Table 1). The majority of vendors were females (n = 342; 55%) as compared to males (n = 275; 45%). The majority of the population (n = 273; 44%) was between the ages of 30 and 39, and in terms of education, the majority of vendors (n = 391; 63%) had completed secondary school, while only 4% (n = 25) had completed tertiary education.

Most cooking vendors also sold non-cooked foodstuff. In terms of non-cooked food, vegetables and fruits were the most popular sold by over 50% (n = 351; 57%) of the informal food vendors, followed by beverages and snacks (n = 76; 12%) and the least sold being eggs, milk and milk products (n = 8; 1,3%). In terms of processed food, cooked meals have been dominating the industry within South Africa as they form part of the country’s stable food. Cooking vendors who dealt with cooked meals accounted for (n= 260; 42%) which included; Pap, rice, relish with a variety of meat, such as chicken or beef meat parts. Fried dough or baked scones were some of the traditional street food served by the vendors in Johannesburg’s inner city (n = 45; 7%), as were cooked maize or mealie (n = 23; 4%); bunny chow (also known as kota in South Africa) and other items which were the least popular, accounting for less than 2% each. 

### 3.3. Exposure Assessment

#### 3.3.1. Static Air Pollution Exposures at Indoor and Outdoor Stalls

CO was found at 0.0 ppm at both indoor and outdoor stalls (Table 2). The PM_2.5_ and CO_2_ concentrations were higher at Site B as compared to Site A. PM_2.5_ and CO_2_ recorded 0.01 mg/m^3^ and 366 ppm at site A (indoor market stall) and 0.07 mg/m^3^ and 397 ppm at site B (outdoor market stall), respectfully. NO_2_ levels per hour between Site A and Site B varied significantly as they differed by a whole 104 μg/m^3^ with the hourly rate for Site A being 55 μg/m^3^ and Site B being extremely higher with a measurement of 159 μg/m^3^. Although not at the same magnitude as the NO_2_ levels, the SO_2_ levels continued the same pattern and were greater at Site B than at Site A. The variation in SO_2_ concentration over 24 hours was just 10 μg/m^3^, with Site B reporting a concentration of 17 μg/m^3^ and Site A reporting a concentration of 7 μg/m^3^ (Table 2).

When comparing the results from Table 2, it is evident that Site B is undoubtedly exposed to higher concentrations than Site A. The higher concentrations at Site B may be emanating from vehicle emissions, offensive trades and construction activities that are evident in inner city Johannesburg. Furthermore, the vendors at Site B are exposed to visible open fires while the indoor market is mostly using electricity and gas for cooking.

#### 3.3.2. Workplace Exposure Related Activities and Personal Behaviors

##### Work Location and Type of Vendor

The majority of the 617 food vendors who participated in the study (n = 338; 55%) worked in indoor food vending sites, while the remainder (n = 279; 45%) worked in outdoor food vending sites. The majority of vendors were cooking vendors and accounted for 348 (56%) in number. Gender roles in socioeconomic development are frequently influenced by cultural orientation and vary geographically. Women in Africa are commonly involved in food preparation and serving; they are taught to cook at a young age. This notion is reflected in the results of this study, which showed that majority of the cooking vendors were females (n = 228; 66%) and the majority of non-cooking vendors were males (n = 155; 58%). Fifty-six percent (n = 348) of the surveyed vendors were exposed to cooking fumes. Informal vendors cook with a variety of heat sources, including gas, wood, and electricity, and each of these heat sources contributes air pollution while cooking. The predominant cooking fuels used by the food vendors were cooking gas (n = 158; 45%), electricity (n = 82; 13%), and open fire (coal and firewood) which accounted for 11% (n = 66). Seven percent (n = 42) of the cooking vendors used more than one type of fuel or a combination of cooking fuel methods.

##### Water Access and Hand Hygiene Practices

The majority of vendors had access to market communal taps (n = 227; 37%), followed by those with taps inside their trading stalls (n = 219; 35%). Those who accessed water from nearby businesses or premises, such as parks, or brought water from home accounted for 27% (n = 164), and lastly those who use other methods, such as buying water for their operations, accounted for 1% (n = 7). The results showed that most indoor vendor markets (204; 93%) have portable water within their stalls as compared to outdoor vendors (n = 15; 7%), and the majority were cooking vendors (n = 176; 80%). Out of the 171 vendors who brought water from home, nearby businesses, or had to buy water, the majority came from outdoor markets (n = 162; 95%).

Those who washed their hands with water and soap for at least 20 s were found to account for 31% (n = 191), while those who sanitized with a 60% alcohol-based sanitizer instead of washing their hands accounted for 32.7% (n = 202). Those who used both washing and sanitising method accounted for 7.8% (n = 48), and 27.9% (n = 172) who practiced hand hygiene without following any standards. With 0.6% (n = 4) of the vendors stating they did not wash their hands, and those four vendors were selling snacks or vegetables. However, it is a struggle for street vendors to practice hand hygiene due to the lack of access to water and hand washing facilities. Only 9.5% (n = 59) vendors practiced hand hygiene at all times, 39% (n = 240) as often as they could, 41% (n = 251) occasionally, and 10% (n = 63) stated to rarely practicing hand hygiene.

##### Respiratory Protective Equipment (RPE) Usage

The results of this study were influenced by COVID-19 regulations; as the study was conducted between the South African Government lockdown mandate of wearing of mask and slightly after the repeal which influenced the usage of RPE. Therefore, vendors who were interviewed between May and June 2022 were mostly adhering to the wearing of masks, while those who were interviewed after June 2022 were mostly not wearing masks. Despite varying usage, surgical masks (n = 356) and cloth masks (n = 256; 41%) were the most common types of masks that vendors had, while N95 masks (n = 5; 0.8%) were the least common. Frequency of using RPE showed that 10% (n = 59) wore their masks at all times, 42% (n = 256) wore it often, 12% (n = 72) occasionally used their masks and 37% (n = 230) never used their masks. Out of the 59 vendors who wore masks at all times, majority were cooking vendors (n = 34; 58%) in contrast to non-cooking vendors (n =25; 42%); and mostly were from indoor markets (n = 32; 54%). About 49% (n = 302) of the vendors changed, washed, or disinfected their masks daily, 9.4% (n = 58) stated two to three times per week, 3.4% (n = 21) weekly, and 1% (n = 6) every two weeks.. The findings of this study contradict those reported by Noomnual and Shendell in 2017 when COVID-19 did not exist, which revealed a lack of protective respiratory mask usage, with only 3% using respiratory masks on a regular basis and 53% never using them [11]. 

##### Health and Safety Training

According to the results, nearly half of the informal food vendors (n = 268; 43%) had health and safety training, and the majority were trained by the Department of Health (n = 255; 41%). However, the bulk of the trained vendors stated that they were only trained once (n = 193; 31%) and received no follow-up training. At least 5% (n = 28) of the informal food vendors received training twice a year, followed by those trained quarterly (n = 16; 3%); those who were trained daily and once a year accounted for 2% (n = 13), respectively, and 0.6% (n = 4) were trained monthly and 0.5% (n = 3) weekly. The majority of the trained vendors (n = 201; 75%) were from indoor markets and were mostly cooking vendors (n = 173; 65%).

#### 3.3.3. Personal Behaviors and Other Exposures

##### Smoking Behaviour

The majority of informal food vendors (n = 477; 77%) were non-smokers, with 38% (n = 181) being males and 62% (n = 296) being females. The majority of the current smokers (n = 111; 18%) and ex-smokers (n = 29; 5%) were male vendors. The results showed that males are disproportionately associated with present smoking habits and a non-smoking habit is disproportionately associated with the female gender.

##### Air Pollution Exposure at Home

The findings in this study indicate that vendors are exposed to air pollutants both at work and at home, which may contribute to an increase in the risk of respiratory health problems. Living close to busy roads, cooking at home, and being close to large industrial sources have all been noted as potential sources of air pollution. The majority of vendors (n = 362; 59%) cooked at home and most of them (n = 220; 61%) were females. The second home air pollution exposure was traffic near homes (n = 156; 25%), followed by those living near a large industrial source (n = 51; 8%).

#### 3.3.4. Work Duration and Frequency of Exposure

The majority of vendors (n = 256; 41%) have worked between 6 and 10 years, followed by those who have worked between 0 and 5 years (n = 216; 35%), those between 11 and 20 years (n = 120; 19%), and only 4% (n = 25) have worked over 20 years (Table 3). According to this study’s results, more than half of the food vendors who worked for ten years and more (n= 84; 58%) were from indoor markets, showing that indoor vendors occupy their stalls longer than outdoor vendors. According to the current study, the majority of the informal food vendors worked over the recommended 8 h a day (n = 451; 73%), with the majority of food vendors trading 6 to 7 days a week (n = 558; 90%). The majority of the vendors who worked more than eight hours a day (n = 251; 56%) were from indoor markets. Furthermore, the majority of vendors (n = 356; 58%) stated that they did not take a break during the working day. Taking breaks during trading hours was mostly adopted by indoor vendors (n = 212; 60%) compared to outdoor vendors (n = 144; 40%).

### 3.4. Prevalence of Respiratory Symptoms and Diseases

The symptom results showed that upper respiratory symptoms (cold, sore throat, and nasal congestion) were the most experienced symptoms by informal food vendors, the highest being a cold at 43%. They were followed by lower respiratory symptoms (coughing, phlegm, breathlessness, and wheezing), the highest being coughing at 39%. Coughing was mentioned as the primary symptom by the majority of cooking vendors (n = 168; 69%), which is similar to a study conducted in a Brunei Darussalam hawker center which stated that coughs were the primary symptom for the majority of cooking vendors (28.3%).

Other symptoms reported included fever, headache, dizziness, fatigue, and eye irritation (with the highest being fever at 34%). Out of 348 cooking vendors, 62% experienced frequent eye, nose, and throat irritation while cooking (Table S1). The fever symptom is associated with the cold symptom and both symptoms were recorded frequently in the results. Fever (34%), headache (26%), and fatigue (15%) were reported to be much more common than in previous studies. These results were in contrast to studies conducted in Bangkok, which both recorded headache and fatigue as the highest experienced symptoms; one study reported 4.49% for headache and 3.46% for fatigue [10], and another one which reported proportions of 20% experiencing headaches and 37% experiencing fatigue [11].

In describing the conditions of the four examined lower respiratory symptoms in the previous 12 months, the results revealed that 152 (25%) vendors cough first thing in the morning in winter, 58 (9%) during the day or at night in winter, and 6 (1%) cough most days for up to three months each year. In terms of the phlegm symptom, 73 (12%) usually bring up phlegm in the morning in winter, 21 (3.4%) during the day or at night in winter, and only 2 (0.3%) bring up any phlegm on most days for up to three months each year. Only four (0.6%) vendors have had a period of increased coughing and phlegm lasting three weeks or lasting three weeks or more in the last three years. In terms of breathlessness, the findings revealed that 5% (n = 31) of respondents experience shortness of breath when hurrying on level ground or walking up a slight hill, 17 (2.8%) experience shortness of breath when walking with others their own age on level ground, and 8 (1%) vendors must stop for breath when walking at their own pace on level ground. The wheezing description of symptoms revealed that 30 (5%) have had attacks of shortness of breath with wheezing, 13 (2.1%) were breathing absolutely normal between attacks, and 4 (0.6%) have been woken up at night due to an attack of shortness of breath.

Nine chest illnesses (1.5%) were reported; bronchial asthma (n = 5; 0.8%) was the highest, followed by 0.2% (n = 1) of each of these illnesses; heart problem, bronchitis, pulmonary tuberculosis, and hay fever resulting in asthma. Most of the vendors with chest illnesses were females (n = 7; 78%), most being cooking vendors (n = 5; 56%) and located at outdoor or street markets (n = 6; 67%) (Table S2). In any of the reported chest illnesses, 44% (n = 4) of the vendors mentioned bringing up more phlegm than usual, while all nine reported having more than 1 illness in the past three years. Due to the study’s timing, which coincided with the historic COVID-19 pandemic, the vendors also reported on the medical diagnosis of COVID-19, which revealed that 105 (17%) vendors contracted COVID-19 since the onset of the pandemic. The indoor informal food vendors (n = 71; 67%) outnumbered outdoor vendors (n = 34; 33%) with COVID-19 diagnoses, which is not surprising due to indoor air quality being a significant factor in the development of the illness.

According to the findings, 318 (52%) of the 617 informal food vendors who participated in the study had a general medical check-up in the previous 12 months, while 299 (48%) had not. Yearly medical attendance was significantly higher for female vendors (n = 194; 61%) than for male vendors (n = 124; 39%). Furthermore, indoor vendors’ attendance to medical check-ups were higher (n = 192; 60%) compared to those who are outdoor vendors (n = 126; 40%). These results may be due to reasons such as that it is extremely difficult for vendors to leave their trading stalls during operational hours for a visit to the clinic as it will cause a loss of attendance of clients for the day, which will have a financial impact.

### 3.5. Risk Characterisation and Consequencess Assessment

#### 3.5.1. Air Pollutants Comparison to Relevant Exposure Limits or Standards

All the air pollutants concentrations for Site A (indoor) and Site B (outdoor) were below the South African [21,22] and WHO legislation levels [23,24,25] (Table 2). CO and CO_2_ concentrations in both sites were far from the recommended limits and there were no major differences in exposure [21,25]. The PM_2.5_ concentrations for Site B (0.07 mg/m^3^) was over 10% of the national OEL (5 mg/m^3^). The NO_2_ level per hour for indoor stall (55 μg/m^3^) and outdoor stall (159 μg/m^3^) were all below the recommended limits of 200 μg/m^3^. However, the following were identified with NO_2_ concentrations at both sites; the NO_2_ concentration at Site B (outside stall) was quite close to the recommended limit [22,24] with a difference of just 41 μg/m^3^ (above 75% of the set guideline value), in contrast to Site A (indoor stall) concentration, which had a difference of 145 μg/m^3^ with the recommended limit. SO_2_ per 24 h concentration at the outdoor market (17 μg/m^3^) and indoor stall (7 μg/m^3^) were all below the South African recommended limits of 125 μg/m^3^ and WHO air quality level of 40 μg/m^3^ [22,23]. The local recommended limit for SO_2_ is about three times higher than the WHO air quality guideline [22,23]. Therefore, when comparing the concentrations to WHO guidelines, the highest concentration (17 μg/m^3^) recorded at Site B (outside stall) was 23 μg/m^3^ less than the guideline value (43% of the set guideline value) compared to the concentration found in Site A (indoor Stall) which was only 33 μg/m^3^ less than the guideline value (18% of the set guideline value).

#### 3.5.2. The Association between Identified Risk Factors to Respiratory Health Symptoms Amongst Informal Food Vendors

**Age:** A positive association was found between age and phlegm (*p* = 0.013), breathlessness (*p* < 0.001), wheezing (*p* = 0.046), headache (*p* = 0.025), dizziness (*p* = 0.003), and fatigue and eye irritation (*p* < 0.001). However, no significant association was found with cold (*p* = 0.112), sore throat (*p* = 0.083), nasal congestion (*p* = 0.121), coughing (*p* = 0.987), and fever (*p* = 0.096).

**Gender:** A positive association was found between gender and sore throat (*p* = 0.033), coughing and phlegm (*p* < 0.001) and wheezing (*p* = 0.023), and all other symptoms, e.g., fever (*p* = 0.003), headache, dizziness, and fatigue and eye irritation (*p* < 0.001). However, no significant association was found with cold (*p* = 0.167), nasal congestion (*p* = 0.115), and breathlessness (*p* = 0.081).

**Type of work location (indoor and outdoor)**: A positive association was found between the type of work location (indoor/outdoor) with all the upper respiratory symptoms (cold, sore throat, and nasal congestion) at *p* < 0.001; coughing and phlegm (*p* < 0.001) and breathlessness (*p* = 0.014); other symptoms, e.g., fever, headache, and dizziness were found at *p* < 0.001. No association was found with wheezing (*p* = 0.078), fatigue (*p* = 0.116), and eye irritation (*p* = 0.479).

**Type of vendor (cooking and non-cooking**): No association was found between the type of vendor (indoor/outdoor) and all upper respiratory symptoms; cold (*p* = 0.199), sore throat (*p* = 0.286), and nasal congestion (*p* = 0.131); lower respiratory symptoms, e.g., phlegm (*p* = 0.189) and breathlessness (*p* = 0.936); and other symptoms, e.g., fever (*p* = 0.156), headache (*p* = 0.211), and dizziness (*p* = 0.226). A positive association was found between the type of vendor (cooking/non-cooking) and coughing (*p* < 0.001), wheezing (*p* = 0.031), fatigue (*p* = 0.017), and eye irritation (*p* = 0.020).

**Type of cooking fuel/heat used:** The type of cooking fuel used was statistically associated with all upper, lower, and other respiratory symptoms. Lower respiratory symptoms were found as a cold (*p* = 0.001), sore throat (*p* = 0.003), and nasal congestion (*p* < 0.001); lower respiratory symptoms were coughing and phlegm at *p* < 0.001, breathlessness (*p* = 0.002), and wheezing (*p* = 0.009); other symptoms were found as fever (*p* = 0.001), headache, dizziness and fatigue at *p* < 0.001, and eye irritation (*p* = 0.033).

**Duration of work (years, days, and hours):** Work duration was found to be statistically significant to all upper respiratory symptoms.

A significant association was found between work duration in years and all upper respiratory symptoms, e.g., cold (*p* = 0.008), sore throat (*p* = 0.015), nasal congestion (*p* = 0.005), coughing (*p* < 0.001), and breathlessness (*p* = 0.008); other symptoms, e.g., headache (*p* = 0.012), dizziness (*p* = 0.041), fatigue (*p* = 0.003), and eye irritation (*p* = 0.002). No association was found with phlegm (*p* = 0.128), wheezing (*p* = 0.067), and fever (*p* = 0.097).

A significant association between work duration in days and all upper respiratory symptoms was found; cold and sore throat at *p* < 0.001, nasal congestion at *p* = 0.001, fever (*p* = 0.006), headache (*p* = 0.003), and dizziness (*p* = 0.022). No association with all lower respiratory symptoms was found; coughing (*p* = 0.920), phlegm (*p* = 0.269), breathlessness (*p* = 0.982), wheezing (*p* = 0.841), fatigue (*p* = 0.062), and eye irritation (*p* = 0.917).

Working hours, showed a statistically significant association with all upper respiratory symptoms, cold, sore throat, and nasal congestion at *p* < 0.001, lower respiratory symptoms (cough and phlegm at *p* < 0.001), and other symptoms; fever (*p* = 0.006) and headache (*p* = 0.004). However, there was no significant association with breathlessness (*p* = 0.296), wheezing (*p* = 0.139), dizziness (*p* = 0.174), fatigue (*p* = 0.348), and eye irritation (*p* = 0.874). 

**Health and safety training:** There was statistical significance between the vendors training and all upper and lower respiratory symptoms; cold (*p* = 0.003), sore throat (*p* = 0.021), nasal congestion (*p* = 0.023), coughing (*p* < 0.001), phlegm (*p* = 0.031), breathlessness (*p* = 0.002), and wheezing (*p* < 0.001). There was also an association with fatigue (*p* = 0.004) and eye irritation (*p* = 0.014). There was no association with fever (*p* = 0.875), headache (*p* = 0.516), and dizziness (*p* = 0.162).

**Frequency of hand hygiene practice at work:** There was a statistical significance between the frequency of hand hygiene practice and all upper and lower respiratory symptoms; cold, sore throat, and nasal congestion at *p* < 0.001, coughing (*p* < 0.001), phlegm (*p* = 0.056), breathlessness (*p* = 0.056), wheezing (*p* = 0.022), and fever (*p* < 0.001). There was no association with headache (*p* = 0.542), dizziness (*p* = 0.839), fatigue (*p* = 0.854), and eye irritation (*p* = 0.891).

**Frequency of using masks at work:** There was a statistical significance between the frequency of using masks at work and all upper respiratory symptoms; cold, sore throat, and nasal congestion at *p* < 0.001, coughing and phlegm at *p* < 0.001, wheezing (*p* = 0.051), and fever, headache, and dizziness (*p* < 0.001). There was no association with fatigue (*p* = 0.063), eye irritation (*p* = 0.060) and breathlessness (*p* = 0.209).

## 4. Discussion

The study’s predominance of female food vendors (55%) is not surprising, as numerous studies have shown that female vendors, particularly in food vending, are the majority [2,3,4,5,6,11,12,13,14,15]. The majority of vendors were between 30 and 39 years; unemployment rates in this age range of 30 to 44 years are typically high [20]. As a result, people in this age group are more likely to use alternative economic options to the formal economy. Furthermore, the age range is consistent with other studies on street vendors, which found that a significant proportion of their respondents fell within a similar age range [2,3,4,5,6,11,12,13,14,15]. 

The majority of the currently smoking vendors were males; the majority of vendors who cook at work and home were females, which increases their risk of respiratory symptoms and diseases. These findings of smoking being the highest amongst males are consistent with other studies conducted in other cities around the world. All of the female street food vendors in Mansoura, Egypt, were non-smokers [26]. However, people who do not smoke, but are exposed to secondhand smoke, are inhaling many of the same cancer-causing substances and poisons that are inhaled by people who smoke [27]. Moreover, since the results of this current study showed that female vendors have experienced the most passive smoking, it will increase the risk of antenatal health complications for female vendors [11,27,28]. Results of a cross-sectional study in Accra, Ghana indicated an association between street vending during pregnancy and birth weight of the newborn, with the average birth weight of babies delivered among street vendors being 177 g (95% CI: 324, 31) lower than babies of mothers of a control group [28].

According to research, there is heavy traffic during rush hours in and around core business centers [29], which may have contributed to ill-health. The concentrations of air pollutants PM_2.5_, SO_2_, NO_2_, and CO_2_ in the outside vendor market area were higher than those at the indoor market area. NO_2_ concentration at Site B (outdoor stall) was only 41 μg/m^3^ less than the guideline values of South Africa and WHO (75% of the set standard), much higher than that of Site A (indoor stall). These results were similar to various other studies. In comparison to residential work areas, roadside work areas had greater concentrations of PM_2.5_, SO_2_, and NO_2_, according to a street vendor study carried out in Bangkok [14]; and in Malaysia, roadside vendors had higher PM_2.5_ and CO levels than indoor restaurant workers [30]. For this study, the chemical exposures assessed at the two sites revealed higher exposures of PM_2.5_, CO_2_, NO_2_, and SO_2_ at the outdoor vendor site compared to the indoor vendor site; these exposures may be related to the prevalence of the respiratory health symptoms and diseases amongst vendors. There is various literature published on possible health risk associated with outdoor workers in relation to the identified air pollutants [12,13,14,15,30,31]. A study in central Italy found that residents’ higher exposure to traffic related air pollutants, PM and NO_2_, had a higher prevalence of coughing with phlegm [31].

Only 1% (n = 9) of the population sampled had current chest illnesses reported, with bronchial asthma being the most reported (n = 5; 0.8%), which were mostly reported by females. These findings are consistent with a study on the effects of Egyptian street vendors’ respiratory systems, which indicated that chronic bronchitis and bronchial asthma were the two most common diseases among female street vendors [14]. Respiratory symptom results showed that the upper symptoms were experienced the most (over 40%), followed by lower respiratory symptoms. These results were consistent with the ones of a current study in Cameroon where upper respiratory symptoms dominated the responses [32]. However, Noomnual and Shendell (2017) reported that lower respiratory illnesses were more common (50%) than upper respiratory illnesses in Bangkok street vendors [15].

There was an identified relationship between work conditions and respiratory health symptoms problems. The risk factors, such as the type of work location, duration, cooking fuels, vendor training, frequency of hand hygiene practice, and frequency of using a mask, were found statistically significant with the upper respiratory symptoms. The evaluation of the work environment is a critical component in protecting workers’ health and controlling contaminant levels at the source. The results of this study showed that outdoor vendors experience more exposures that are detrimental to health compared to indoor vendors. The findings of this study are consistent with those of a study carried out in Johannesburg by Kalitanyi (2021), which found that there is a lack of infrastructure and temporary structures for outdoor or street vendors to sell their items [33]. The observation of poor sanitary conditions in the majority of the food vending sites is similar to those reported in a Kenyan study where it was observed that about 85.0% of the vendors prepared their food in unhygienic condition [34]. However, opposite findings were found in other African countries, such as studies conducted in Nigeria [35] and in Ghana [36], where the majority of the food premises were observed to be tidy, with the use of waste bin and the presence of onsite water source for sanitary purposes. The noise generated by the cars circulating along these highways, as well as public movement in the inner-city walkways, may have contributed to the high rates of fatigue and headaches.

Working duration has proven to be a major risk factor in occupational health [18,21,37]. This study found that the majority of the vendors work over the recommended 8 h a day (73%) and 6 to 7 days a week (90%), and only a little over 50% of the vendors took a break during trading hours (58%); opposing the ILO guidelines [18]. The majority of the vendors worked between 6 to 10 years (41%). In the city of Douala, Cameroon, vendors worked over 8 h (92.2%). In Mansoura, Egypt, 92.8% of the studied street vendors worked for more than 8 h daily, more than half (55.9%) worked for the entire week, and the majority (89.5%) did not take any rest breaks during the working day [26]. The results of indoor vendors trading more than eight hours could be for the reason that indoor markets are perceived to be permanent, with indoor storage facilities and usually have a manager and security and may experience lower criminality. While outdoor vendors are trading independently far from their storage areas and without security personnel. Therefore, the outdoor or street vendors may leave their workplace before sunset. A study in Malaysia demonstrated a positive association between working hours with breathing difficulty (*p* = 0.011) and sputum production (*p* = 0.014) and also between cough and the type of cooking fuel used [13]. Additionally, a systematic review conducted by Wong, Chan, and Ngan (2019) found that workers who worked more than 50 h per week had an increased risk of cerebrocardiovascular diseases, myocardial infarction, and coronary heart disease [38]. 

This study reported a positive association between the type of cooking fuel used with all upper, lower, and other respiratory symptoms, which is similar to findings of other studies. Cooking gas (45%) was the highest cooking fuel used. In a review by Basu and Samet (1999), their conclusion was that NO_2_ emitted by gas stoves is not risk free [39]. Basu and Samet (1999) further stated that indoor use of gas stoves was linked with lower respiratory symptoms (OR = 1.23; 95% CI = 1.03, 1.47) and with chronic respiratory conditions (OR = 2.08; 95% CI = 1.49, 2.90) [39]. However, these results are opposite to Wahid et al. (2014) whose results discovered that those who cooked with charcoal were two times more likely to cough than those who cooked with liquefied petroleum gas [40]. This study’s findings showed that coughing was the primary symptom of cooking vendors (n = 168; 69%) and there were similar results from Malaysia of significant association between the type of cooking fuel used and the presence of coughing (*p* = 0.001) [35], and a study in Brunei Darussalam reported coughing reported by 50% of the food vendors who had been in business for more than ten years, and it was twice as prevalent among charcoal cooks as it was among LPG cooks [40].

Even though the air pollutants concentrations were found to be below national and international standards, there have been reports that the present respiratory disease prevention and management in the vending trade are insufficient. This is also shown by the findings of unsanitary vending site conditions and lack of infrastructure which are non-compliant to the Occupational Health and Safety (OHS) Act (No. 85 of 1993) [37], and the Regulations Governing General Hygiene Regulations for Food Premises, the Transport of Food, and Related Matters’ (no. R638 of 22 June 2018) requirements (no. 85 of 1993) [41]. The majority of vendors accessed water from communal taps (37%). Due to poor hand-hygiene habits and unsanitary vendor stalls, street vendors who rely on shared water sources are more susceptible to illnesses linked to general hygiene, such as respiratory problems [16]. This study’s findings also showed that incorrect RPE was used (most vendors used surgical and cloth masks) which may provide ineffective protection for the traffic related air pollutants; furthermore, there was no difference between participants who used RPE and those who did not use RPE in developing respiratory symptoms [17]. Another key problem that informal vendors face is that, although they have free access to primary health care services, they lose money whenever they leave work for healthcare or other reasons, unlike the workers in formal employment. Therefore, only 52% of the informal food vendors went for a medical check-up in the previous 12 months. Individual behaviors are mostly influenced by knowledge. There was lack of continuous health and safety training; moreover, most indoor vendors are trained compared to outdoor vendors. These results may be due to the permanency of indoor vendor market stalls, which results in vendors staying longer in those stalls compared to outdoor vendors who may stay at their vending space for a short period of time. There is little education provided to vendors regarding air pollution and respiratory health. Since the majority of training is centered on food safety and general health and hygiene [2,3,4,5], the majority of vendors, particularly cooking vendors, are unaware of the health consequences of prolonged exposure to smoke from both indoor and outdoor cooking, which will not only be an individual life impact but impact the entire health system [19,20].

## 5. Conclusions

The health and welfare of those who work in the informal sector is a significant and understudied subject that is covered by this study. The associations between different occupational risks and health outcomes among street vendors have been reported by limited international studies in recent years. Moreover, most air pollution studies concentrate on general human pollution exposures, but limited studies exist on exposure to the pollutants in an occupational setting, especially that of informal workers. As per the knowledge of the authors, this was the first study in South Africa to extensively assess workplace exposure of vendors. The study used a thorough risk assessment procedure that included a walkthrough inspection of vending markets, measuring chemical exposure, and administered a lengthy questionnaire to street vendors in both indoor and outdoor markets. The outcomes of the two populations were then compared.

In addition to an elevated risk to reproductive and cardiovascular health, ambient air pollution has been associated with upper and lower respiratory health problems amongst informal vendors. This study’s results showed that the most experienced symptoms were upper respiratory symptoms, with the possibility of an increase in risk for outdoor market vendors, especially cooking vendors. However, even though the outdoor vendor site had higher exposures of PM_2.5_, CO_2_, NO_2_, and SO_2_, it does not conclude that indoor vendors are risk free.

Various conclusions were drawn from the study. The street vendor’s location, lack of infrastructure, usage of dirty cooking fuels, lack of health and safety knowledge, and practice places them at an increased health risk. There is a lack of sustainable and measurable health and safety training for vendors. There is little education provided to vendors regarding air pollution and respiratory health; the majority of vendors, particularly cooking vendors, are unaware of the health consequences of prolonged exposure to smoke from both indoor and outdoor cooking. The burden of respiratory diseases could lead to an increase in health care costs that could have an impact on the entire health system [14]. Furthermore, the findings of this study show that there is lack of compliance to the health legislations.

The legislature should investigate the current rules and regulations governing informal trade, as well as the local government policies and management challenges. This process will help in identifying the challenges at the implementation level and assist in the development of relevant and suitable legislation for this group. There is also a need for the formalization of this trade, which will entail the improvement of infrastructure (creating the ideal stalls and markets) and should involve all relevant stakeholders, including the vendors’ forums. Respiratory health care services, such as lung function testing, are needed to identify health problems at an early stage. The informal vendors should also comply with legislations and practice general hygiene during operational hours.

## Figures and Tables

**Table 1:** Demographical characteristics of informal food vendors in the inner city of Johannesburg.

Demographic Characteristics	Gender		Total No = 617
	Male = n (%)	Female = n (%)	
Gender	275 (45%)	342 (55%)	617 (100%)
**Nationality**			
South African	137 (41%)	196 (59%)	333(54%)
Non-south African	138 (49%)	146 (51%)	284 (46%)
**Age Groups (years)**			
18–29	48 (51%)	47 (49%)	95 (15%)
30–39	125 (46%)	148 (54%)	273 (44%)
40–49	88 (44%)	111 (56%)	199 (32%)
50>	14 (28%)	36 (72%)	50 (8%)
**Educational Level**			
Never attended	15 (43%)	20 (57%)	35 (6%)
Primary	89 (54%)	77 (46%)	166 (27%)
Secondary	160 41%)	231 (59%)	391 (63%)
Tertiary/ Higher	11 (44%)	14 (56%)	25 (4%)

**Table 2:** Descriptive statistics for stall daily air pollutants (static sampling) and recommended exposure levels at vendor markets in the inner city, Johannesburg.

Air Pollutant	Indoor (Site A)	Outdoor (Site B)	South African Standards			International Standards		
			Name	Limit	Duration	Name	Limit	Duration
PM_2.5_ (mg/m^3^)	0.01 mg/m^3^	0.07 mg/m^3^	HCA, 2021	5 mg/m^3^	8 h	WHO, 2021	15 μg/m^3^	24 h
SO_2_ (μg/m^3^)	7 μg/m^3^	17 μg/m^3^	NEMA, 2004	125 μg/m^3^	24 h	WHO, 2021	40 μg/m^3^	24 h
NO_2_ (μg/m^3^)	55 μg/m^3^	159 μg/m^3^	NEMA, 2004	200 μg/m^3^	1 h	WHO, 2021	200 μg/m^3^	1 h
CO_2_ (ppm)	366 ppm	397 ppm	HCA, 2021	5000 ppm	8 h	NIOSH, 1994	5000 ppm	8 h
CO (ppm)	0.0 ppm	0.0 ppm	HCA, 2021	4000 ppm	8 h	WHO, 2021	35 μg/m^3^	1 h

**Table 3:** Occupational characteristics of informal food vendors in the inner city of Johannesburg (work duration exposure).

Occupational Characteristics		Work Location		Total No = 617
		Indoor = n (%)	Outdoor = n (%)	
Duration of food Vending/years	0–5 years	101 (47%)	115 (53%)	216 (35%)
	6–10 years	153 (60%)	103 (40%)	256 (41%)
	11–20 years	73 (61%)	47 (39%)	120 (19%)
	>20 years	11 (44%)	14 (56%)	25 (4%)
Working days/week	2 days	1 (100%)	0	1 (0, 2%)
	3 days	2 (67%)	1 (33%)	3 (0, 5%)
	5 days	26 (47%)	29 (53%)	55 (8, 9%)
	6 days	174 (59%)	121 (41%)	295 (47, 8%)
	7 days	135 (51%)	128 (49%)	263 (42, 6%)
Working hours/week	<8 h	34 (63%)	20 (37%)	54 (9%)
	8 h	53 (47%)	59 (53%)	112 (18%)
	>8 h	251 (56%)	200 (44%)	451(73%)
Break during the working day	Yes	212 (60%)	144 (40%)	356 (58%)

## Data Availability

All data in this study were provided in the main manuscript.

## Supplementary Materials

The following supporting information can be downloaded at: https://www.mdpi.com/article/10.3390/ijerph20032736/s1, Table S1: Prevalence of upper and lower respiratory symptoms and diseases among informal food vendors in the inner city of Johannesburg; Table S2: Prevalence of respiratory chronic diseases or illnesses among informal food vendors in the inner city of Johannesburg.
