# Supplementary material for: Health Risk Assessment of Informal Food Vendors: A Comparative Study in Johannesburg, South Africa
Source: Int J Environ Res Public Health. 2023 Feb 3;20(3):2736. doi: 10.3390/ijerph20032736 (PMC9915406; doi:10.3390/ijerph20032736)
Supplement: Supplementary file 1 [file ijerph-20-02736-s001.zip › ijerph-2140608-supplementary.pdf]

Table S1. Prevalence of upper and lower respiratory symptoms and diseases among informal food vendors in the inner city of Johannesburg.

Respiratory problems		Total No=617	Work location		Type of vendor		Gender	
			Indoor	Outdoor	Cooking vendors	Non-cooking vendors	Male	Female
			N (%)	N (%)	N (%)	N (%)	N (%)	N (%)
**Upper symptoms in the last 12 months**	Cold	268 (43%)	114 (43%)	154 (57%)	159 (59%)	109 (41%)	111 (41%)	157 (59%)
	Sore throat	258 (42%)	109 (42%)	149 (58%)	152 (59%)	106 (41%)	102 (40%)	156 (60%)
	Nasal congestion	248 (40%)	106 (43%)	142 (57%)	149 (60%)	99 (40%)	101 (41%)	147 (59%)
**Lower symptoms in the last 12 months**	Cough	242 (39%)	110 (46%)	132 (54%)	168 (69%)	74 (31%)	77 (32%)	165 (68%)
	Phlegm	125 (20%)	44 (35%)	81 (65%)	77 (62%)	48 (38%)	37 (30%)	88 (70%)
	Breathleness	28 (5%)	9 (32%)	19 (68%)	16 (57%)	12 (43%)	8 (29%)	20 (71%)
	Wheeziness	43 (7%)	18 (42%)	25 (58%)	31 (72%)	12 (28%)	12 (28%)	31 (72%)
**Cough**	Usually cough 1st thing in the morning in winter	152 (25%)	67 (44%)	85 (56%)	103 (68%)	49 (32%)	42 (28%)	110 (72%)
	Usually cough during the day or at night in the winter	58 (9%)	31 (53%)	27 (47%)	44 (76%)	14 (24%)	17 (29%)	41 (71%)
	Cough like this on most days for as much as three months each year	6 (1%)	1 (17%)	5 (83%)	4 (67%)	2 (33%)	0	6 (100%)
**Phlegm**	Usually bring up any phlegm in the morning in winter	73 (12%)	20 (27%)	53 (73%)	42 (58%)	31 (42%)	17 (23%)	56 (77%)
	Usually bring up any phlegm during the day or at night in the winter	21 (3, 4%)	6 (29%)	15 (71%)	18 (86%)	3 (14%)	6 (29%)	15 (71%)
	bring up any phlegm like this on most days for as much as three months each year	2 (0, 3%)	0	2 (100%)	1 (50%)	1 (50%)	1 (50%	1 (50%)
	in the past 3 years have you had a period of increased cough and phlegm lasting for 3 weeks or more	3 (0, 5%)	0	3 (100%)	2 (67%)	1 (33%)	0	3 (100%)
	in the past 3 years have you had a period of increased cough and phlegm lasting for 3 weeks or more, more than 3 winters)	1 (0, 2%)	0	1 (100%)	0	1 (100%)	0	1 (100%)
**Breatheleness**	troubled by shortness of breath when hurrying on level ground or walking up a slight hill	31 (5%)	5 (16%)	26 (84%)	19 (61%)	12 (39%)	7 (23%)	24 (77%)
	Short of breath when walking with others of your own age on level ground	17 (2, 8%)	2 (12%)	15 (88%)	12 (71%)	5 (29%)	4 (24%)	13 (76%)
	Stops for breath when walking at your own pace on level ground?	8 (1%)	1 (12%0	7 (88%)	6 (75%)	2 (25%)	1 (12%)	7 (88%)
**Wheeziness**	Ever had attacks of shortness of breath with wheezing	30 (5%)	13 (43%)	17 (57%)	23 (77%)	7 (23%)	7 (23%)	23 (77%)
	Is/was your breathing absolutely normal between attacks? (Yes)	13 (2.1%)	5 (38%)	8 (62%)	9 (69%)	4 (31%)	3 (23%)	10 (77%)
	At any time in the last 12 months been woken up at night by any attack of shortness of breath	4 (0, 6%)	0	4 (100%)	3 (75%)	1 (25%)	1 (25%)	3 (75%)
**Other acute symptoms**	Fever	207 (34%)	88 (43%)	119 (57%)	125 (60%)	82 (40%)	75 (36%)	132 (64%)
	Headache	160 (26%)	65 (41%)	95 (59%)	97 (60%)	63 (40%)	46 (29%)	114 (71%)
	Dizziness	107 (17%)	40 (37%)	67 (63%)	66 (62%)	41 (38%)	32 (305)	75 (70%)
	Fatigue	93 (15%)	44 (47%)	49 (53%)	63 (68%)	30 (32%)	24 (26%)	69 (74%)
	Eye irritation	50 (8%)	25 (50%)	25(50%)	36 (72%)	14 (28%)	10 (205)	40 (80%)
	Frequency of eye, nose and throat irritation while cooking — Total who experienced irritation	**124 (37%)** out of 348 cooking vendors	**47** (22%) out of the 210 indoor cooking vendors	**77 (56%)** out of the 138 outdoor cooking vendors	**124(37%)** out of 348 cooking vendors	**N/A**	**38 (32%)** out of the 120 cooking males vendors	**86 (38%)**out of the 228 cooking females vendors
		**% of total**	**40%**	**62%**	**100%**	**N/A**	**31%**	**69%**
	Never	215 (62%)	160 (74%)	55 (26%)	215(100%)	N/A	78 (36%)	137 (64%)

	Rarely	56 (16%)	19 (34%)	37 (66%)	56(100%)	N/A	21 (38%)	35 (63%)
	Sometimes	62 (18%)	26 (41%)	36 (59%)	62(100%)	N/A	17 (27%)	45 (73%)
	Often	4 (1%)	1 (25%)	3 (75%)	4 (100%)	N/A	0	4 (100%)
	Always	2 (0.6%)	1 (50%)	1 (50%)	2 (100%)	N/A	0	2 (100%)

Table S2. Prevalence of respiratory chronic diseases or illnesses among informal food vendors in the inner city of Johannesburg.

Respiratory problems		Total No=617	Work location		Type of vendor		Gender	
			Indoor	Outdoor	Cooking vendors	Non-cooking vendors	Male	Female
			N (%)	N (%)	N (%)	N (%)	N (%)	N (%)
**Chest illness in the last past three years**		**9 (1, 5%)**						
Which chest illness kept you from usual activities for as much as a week or longer	Heart trouble	1 (0, 2%)	1 (100%)	0	1 (100%)	0	0	1 (100%)
	Bronchitis	1 (0, 2%)	0	1 (100%)	0	1 (100%)	0	1 (100%)
	Pulmonary tuberculosis	1 (0, 2%)	0	1 (100%)	0	1 (100%)	0	1 (100%)
	Bronchial Asthma	5 (0, 8%)	2 (40%)	3 (60%)	3 (60%)	2 (40%)	2 (40%)	3 (60%)
	Hay fever resulting in Asthma	1 (0, 2%)	0	1 (100%)	1 (100%)	0	0	1 (100%)
Other related information	Bring up phlegm more than usual in any of the illness	4 (0, 6%)	0	4 (100%)	2 (50%)	2 (50%)	0	4 (100%)
	Had more than 1 illness like this in the past 3 years	9 (1,5%)	3 (33%)	6 (67%)	5 (56%)	4 (44%)	2 (22%)	7 (78%)
**Past medically diagnosed diseases**		**8 (1,3%)**						
Past illnesses: Have you ever been Medically diagnosed with any of these diseases?	Bronchitis	1 (0, 2%)	0	1 (100%)	0	1 (100%)	0	1 (100%)
	Pulmonary tuberculosis	2 (0,3%)	1 (50%)	1 (50%)	0	2 (100%)	1 (50%)	1 (50%)
	Bronchial Asthma	4 (0, 6%)	1 (25%)	3 (75%)	3 (75%)	1 (25%)	1 (25%)	3 (75%)
	Hay fever resulting in Asthma	1 (0, 2%)	0	1 (100%)	1 (100%))	0	0	1 (100%)
Other illnesses	Covid-19 between 2020-2022	105 (17%)	70 (67%)	35 (33%)	46 (44%)	59 (56%)	46 (44%)	59 (56%)
